# Modified Dual Hepatic Vein Anastomosis in Pediatric Living-Donor Liver Transplantation Using Left Lateral Segment Grafts With Two Wide Orifices

**DOI:** 10.3389/fped.2021.685956

**Published:** 2021-09-17

**Authors:** Yuchen Hou, Ping Wan, Mingxuan Feng, Bijun Qiu, Tao Zhou, Jianjun Zhu, Yi Luo, Jianjun Zhang, Qiang Xia

**Affiliations:** Department of Liver Surgery, School of Medicine, Ren Ji Hospital, Shanghai Jiao Tong University, Shanghai, China

**Keywords:** outflow obstruction, left hepatic vein, anatomical variations, LLS, conduit

## Abstract

**Background:** The anatomic variation of hepatic vein in the left lateral segment (LLS) increases the risk of outflow complication in pediatric living liver transplantation (LDLT). Here, we share a modified method for dual hepatic vein reconstruction in pediatric LDLT using LLS with two wide orifices.

**Methods:** From Sep 2018 to Dec 2019, 434 pediatric LDLTs using LLS were performed in our center. Hepatic veins of grafts were classified into three types with emphasis on the number, size, and location of orifices at the cut surface: a single opening (type I, *n* = 341, 78.57%); two adjacent orifices (type II, *n* = 66, 15.21%); two wide orifices with orifices distances <20 mm (type IIIa, *n* = 15, 3.46%); and two wide orifices with orifices distances >20 mm (type IIIb, *n* = 12, 2.76%). Rv was defined as the ratio of diameter of V2 and V3 (refer to hepatic vein drained segments II and III). We developed a modified dual hepatic vein anastomosis to reconstruct outflow for type IIIb grafts with Rv ≤1. Briefly, the hepatic vein of segment II was anastomosed to the common stump of middle hepatic vein (MHV) and left hepatic vein (LHV), followed by unification of V3 and the longitudinal incision orifice in inferior venous cave (IVC).

**Results:** During median follow-up of 15.6 months (7.5–22.9 months), no hepatic vein complications occurred.

**Conclusion:** This novel modified dual hepatic vein anastomosis could serve as a feasible surgical option for type IIIb LLS grafts with Rv ≤1 in pediatric LDLT.

## Introduction

Liver transplantation is the standard choice for children with end-stage liver diseases (1). Due to the organ shortage of deceased donors, living-donor liver transplantation (LDLT) has become a widely accepted therapeutic option (2). Pediatric liver transplantation account for 7–8% of total number of liver transplants, and 50–80% of transplants are done at <3 years of age (1). The left lateral segment (LLS) grafts from living donors, which represents 15–20% of donors' total liver mass, is most frequently used in infants or small children (3). In most cases, LLS grafts only have a single hepatic vein orifice in cut surface which is anastomosed end-to-end to the left of the common stump of MHV and LHV (4). However, anatomical variations of hepatic veins in LLS is common and two wide HV orifices account for 2–4% of all LLS grafts (5, 6). Adequate hepatic venous outflow is essential for survival of graft and patients (7–9). For LLS grafts with two wide hepatic vein orifices, it is technically challenging if it occurs (10). Some transplant centers reconstructed utilizing interpositional grafts, especially cryopreserved iliac artery (11). Here, we summarize anatomical LHV variations and describe a modified dual hepatic vein anastomosis technique for LLS with two wide orifices.

## Patients and Methods

### Patients and Characteristics

Four hundred fifty-three LDLT procedures were performed at the Department of Liver Surgery, Renji Hospital, School of Medicine, Shanghai Jiaotong University from September 2018 to December 2019. All LDLT procedures were approved by the ethics committee of Renji Hospital and were performed in accordance with the relevant regulations. A retrospective analysis of left hepatic vein (LHV) variations in LLS grafts was performed with emphasis on the number, size, and location of orifices at the cut surface. Computer tomography image data for 434 donors using LLS were analyzed. The anatomical variations of LHV were classified into three types. Very small hepatic vein branches (<5 mm in diameter) draining into the middle hepatic vein (MHV) or inferior vena cava (IVC) were not considered because they could be sacrificed without the risk of significant hepatic vein congestion. Eight type IIIb LLS grafts were considered fitter for modified dual hepatic vein anastomosis.

### Donor Operation

LLS graft harvest strategies were formulated according to computational simulation of virtual LLS graft with commercial three-dimensional reconstruction software (IQQA-3D; EDDA Technology). Graft/recipient's body weight ratio (GRWR) was calculated, and all 434 donors received left lateral segmentectomy. The hepatic parenchyma was transected using an ultrasonic aspirator (Sonaca; Soring Inc., Quickborn, Germany) and bipolar electric cautery. The triangular and hepatogastric ligaments were dissected, and the hepatic veins were isolated. The left portal vein and left hepatic artery were transected and the sheath around the left bile duct was left undisturbed to maintain the blood flow of the biliary system. The isolated graft was then perfused *in situ* through the left portal vein, first with 4°C lactated Ringer's solution (200 ml), and then with 4°C UW solution (600–1,000 ml).

### Recipient Operation

Hepatic artery and portal vein were isolated at the hepatic hilum, then the liver was dissected from IVC by dissection of ligation and short hepatic veins. Total hepatectomy was performed after side clamping the IVC. The liver graft was implanted into the hepatic cavity by hepatic vein anastomosis. The vascular reconstruction was performed in end-to-end fashion in the portal vein with 7–0 polyglyconate (PDS). The hepatic artery anastomosis was accomplished with 8–0 or 9–0 polypropylene (Prolene) with the surgical microscope (Model S88, Carl Zeiss Inc., Oberkochen, Germany). Biliary reconstruction was performed with a Roux-en-Y limb or interposed jejunal conduit previously existing in patients treated with Kasai portoenterostomy.

### Techniques of Modified Dual Hepatic Vein Anastomosis

**Figure 1:**
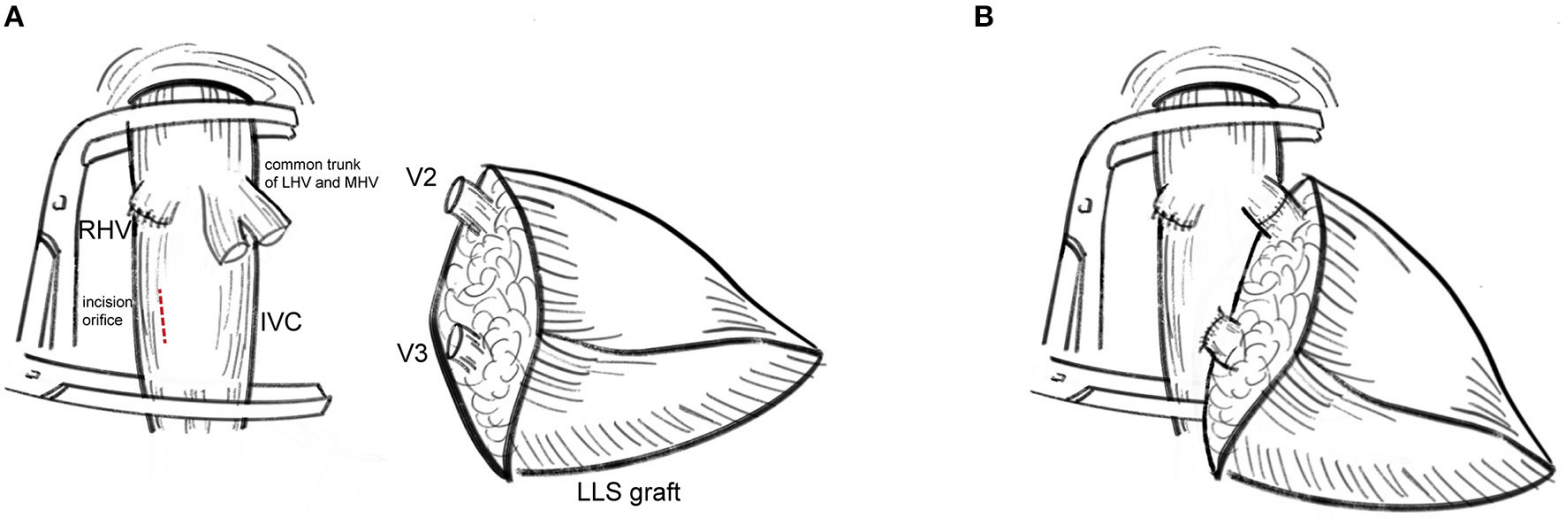
Procurement of the modified dual hepatic vein anastomosis. **(A)** V2 was anastomosed to the stump of MHV and LHV while V3 was anastomosed to the incision orifice at the right of the middle line of IVC. **(B)** The cut surface of liver graft was adjusted to slightly oblique upward to prevent regenerated LLS graft oppress-reconstructed hepatic veins. The modified dual hepatic vein anastomosis leave enough space for graft regeneration.

The modified dual hepatic vein anastomosis technique is a better choice for hepatic vein reconstruction in LLS grafts with two wide orifices (>20 mm) and R_V_ ≤ 1 (V3 as the dominant hepatic vein). The distance between two orifices should smaller than the length of retrohepatic inferior vena cava. As shown in Figure 1, modified dual hepatic vein anastomosis could be accomplished by the following:

1. V2 is anastomosed end-to-end to the left of the common stump of MHV and LHV.
2. An oval incision orifice is created close to the central axis of the IVC in recipients. The diameter of incision orifice should not be smaller than the size of V3. Whereas, V3 is anastomosed end-to-side to the longitudinal incision orifice in the anterior wall of IVC with 5–0 polyglyconate (PDS).

Eversion suture was not suggested to expand outflow size. Care was taken to adjust the cut surface of liver graft slightly oblique upward to avoid liver regeneration-related hepatic vein occlusion.

### Postoperative Management

Doppler ultrasound (US) was used to estimate the outflow status at the completion of hepatic vein reconstruction and the completion of transplantation. We then performed Doppler US tests in all recipients daily in the 1st week after LT, every 2 days in the 2nd week, monthly during the first 6 months, and every 3 months thereafter. Oral administration of FK506 is initially at doses of 0.1–0.15 mg/kg every day. Trough levels of FK 506 were maintained in the range of 8~12 ng/ml for the 1st month and gradually decreased to 5 ng/ml after 1 year. Cyclosporine was initial at a dose of 4 mg/kg/day, and the target C0 and C2 levels were 150–200 and 800–1,200 ng/ml, respectively. CYP3A5 genotypes in both recipients and donors were performed to guide the usage of FK506 and cyclosporine (12). Additional mycoohenolate mofetil was used if FK506 or cyclosporine did not reach the target level. Steroid administration is initial at a dose of 10 mg/kg at anhepatic phase during transplantation and 4 mg/kg/day after operation. The dose of methylprednisolone was then gradually tapered by 4 mg/day and maintained with oral administration of prednisone at 2.5 mg/day. Prednisone was discontinued 3–6 months after LDLT (13). The anticoagulant regimen included intravenous heparin for 2 weeks.

### Statistical Analysis

All data analysis in this research was performed using R software. All results were expressed as mean ± standard error of the mean.

## Results

### Anatomical LHV Variations in LDLT Using LLS Graft

**Figure 2:**
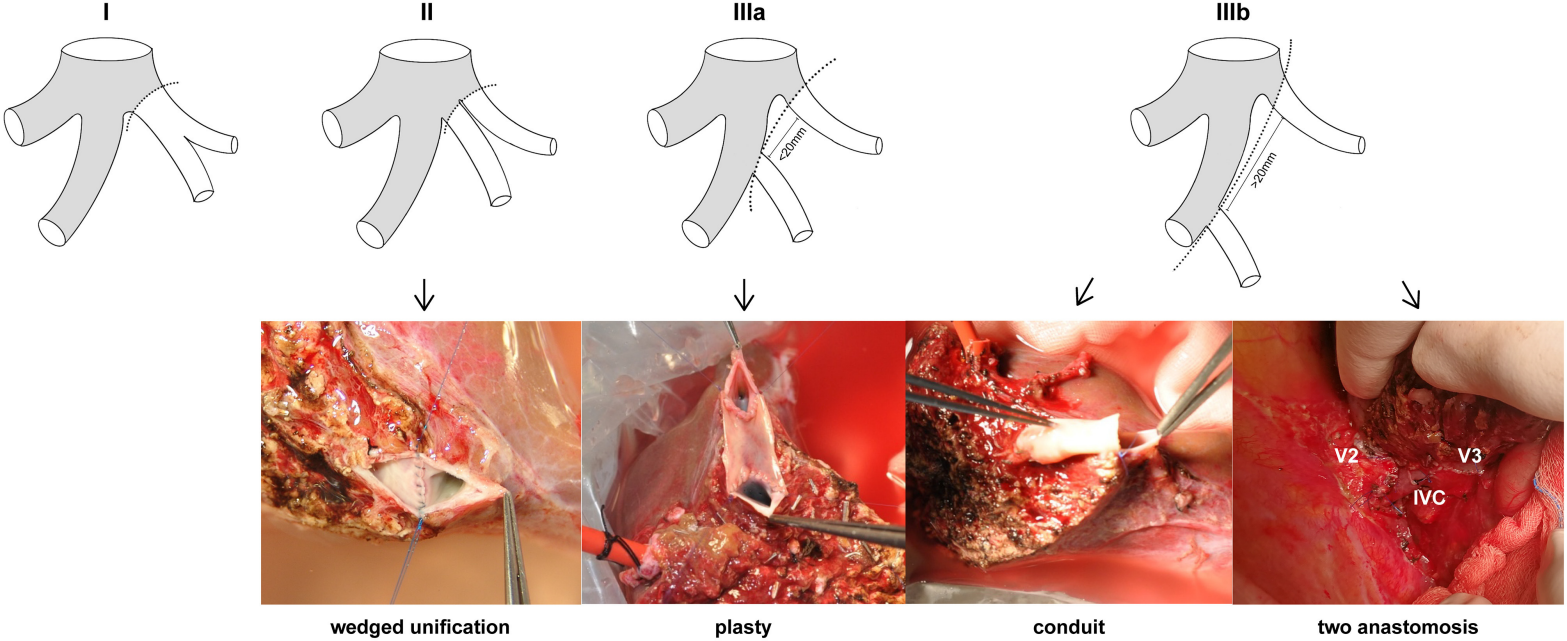
Anatomical variations of LHV in LLS grafts. Type I: a single orifice; type II: two close orifices; type IIIa: two separated orifices (distance <20 mm); type IIIb: two separated orifices (distance >20 mm). Wedge unification is the first choice for type II. Venolasty to reform a common ostium is recommended for type IIIa. Conduit and two independent anastomosis are used for type IIIb.

The LHV anatomy of 434 LLS graft donors was classified on the basis of the patterns of the graft hepatic veins. The mean age of the donors was 31.42 years (range = 18–58 years), and 210 (48.39%) were male. The patterns of the LLS graft hepatic veins were classified into types according to the number, size, and location of orifices at the cut surface (Figure 2): (I) a single opening (*n* = 341, 78.57%); (II) two close orifices (*n* = 66, 15.21%); (III) two wide-spaced orifices (*n* = 27, 6.22%). According to the distance between two separated openings, type III can be classified as: (a) orifices distances <20 mm (*n* = 15, 3.46%) and (b) orifice distances >20 mm (*n* = 12, 2.76%). R_V_ was defined as the ratio of diameter of V2 and V3 which can be used to determine the dominant hepatic vein in LLS. For type IIIb LLS grafts with R_V_ ≤ 1, modified dual hepatic vein anastomosis is preferable to ensure satisfactory hepatic venous drainage.

### Characteristics of Recipients and Donors

A consecutive series of recipients using modified dual hepatic vein anastomosis included four boys and fouor girls with a median age of 22.3 months (range, 6–50 months) and a median weight of 10.2 kg (range, 4.6–17 kg). Four of the children suffered from biliary astresia (BA), and the other four children's primary diseases were acute hepatic failure, hyperhomocysteinemia, tyrosinemia, and ornithine transcarboxylase deficiency (OTCD), respectively. The profile of donors are summarized in Table 1, and the detailed clinical characteristics of each patient accepting modified dual hepatic vein anastomosis are summarized in Table 2.

**Table 1:** Profile of donors for modified dual HV anastomosis.

**Donor**	**Age (years)**	**Sex**	**Height (cm)**	**Weight (kg)**	**BMI of donor**	**Distance between orifices (mm)**	**Dimeter of V2 (mm)**	**Dimeter of V3 (mm)**	**Rv (V2/V3)**	**Relationship between donor and recipient**
1	34	Female	160	47.5	18.55	22.82	6.84	8.78	0.78	Mother
2	27	Female	163	46	17.31	23.41	5.23	6.71	0.77	Mother
3	27	Male	171	88	30.09	23.15	6.44	8.08	0.79	Father
4	30	Male	170	55	19.03	22.83	6.97	8.69	0.80	Father
5	30	Female	165	60	22.04	28.36	5.79	6.63	0.87	Mother
6	32	Male	168	52	18.42	29.57	6.56	7.33	0.89	Father
7	36	Male	168	54	19.1	22.91	7.33	8.06	0.91	Father
8	33	Male	170	70	24.22	29.72	7.21	7.47	0.97	Father

**Table 2:** Individual information for each patient receiving modified dual HV anastomosis.

**Patient**	**Age (months)**	**Sex**	**Weight (kg)**	**Indication for LT**	**Kasai history**	**Graft weight (g)**	**GRWR (%)**	**HV reconstruction time (min)**	**Graft function recovered time (day)**	**Drainage tube indwelling time (days)**	**Hospital stay time (days)**	**Follow-up duration (months)**	**Survival**
1	6	Male	8.9	BA	Y	210	2.4	28	13	11	14	7.5	Alive
2	6	Female	7.3	BA	Y	200	2.7	23	11	11	16	14.7	Alive
3	8	Male	4.6	BA	N	230	5.0	27	11	13	15	17.1	Alive
4	18	Female	7.4	BA	N	195	2.6	37	10	37	37	22.9	Alive
5	39	Female	13	OTCD	N	230	1.8	26	14	11	16	7.7	Alive
6	50	Male	17	ALF	N	320	1.9	28	/	7	19	22.4	Alive
7	19	Male	10	Tyrosinemia	N	185	1.9	39	3	18	24	11.1	Alive
8	33	Female	13.5	HHE	N	255	1.9	31	13	12	14	21.2	Alive

*OTCD, ornithine transcarboxylase deficiency; ALF, acute liver failure; HHE, hyperhomocysteinemia*.

*Graft function recovered time indicate the time from surgery to the day TB, ALT, and AST drop to normal levels*.

### Operational Results

For the eight LDLTs using modified dual hepatic anastomosis, the grafts were all left lateral segments and the median GRWR was 2.5% (1.8–5.0%). The median time for hepatic vein reconstruction was 22.88 min (17–31 min). The median hepatic phase was 47.38 min (44–54 min). The median distance between two orifices was 25.30 mm (22.82–29.57 mm). The median diameter of V2 was 6.55 mm (5.23–7.33 mm), and the median diameter of V3 was 7.72 mm (6.63–8.69 mm). The median R_V_ was 0.85 (0.77–0.97). All of the grafts showed good venous outflow, and the Doppler US results at the completion of transplantation are summarized in Table 3. Detailed information is shown in Table 2.

**Table 3:** Postoperational Doppler ultrasound profile for hepatic vein flow.

**Patient**	**During operation**				**Postoperation day 7**				**Postoperation day 14**			
	**V2**		**V3**		**V2**		**V3**		**V2**		**V3**	
	**Wave pattern**	**Vmax (cm/s)**	**Wave pattern**	**Vmax (cm/s)**	**Wave pattern**	**Vmax (cm/s)**	**Wave pattern**	**Vmax (cm/s)**	**Wave pattern**	**Vmax (cm/s)**	**Wave pattern**	**Vmax (cm/s)**
1	Biphasic	44	Biphasic	32	Monophasic	30	Biphasic	34	Monophasic	55	Biphasic	42
2	Biphasic	22	Monophasic	26	Biphasic	28	Biphasic	29	Biphasic	44	Biphasic	46
3	Biphasic	32	Biphasic	36	Biphasic	42	Biphasic	44	Biphasic	46	Biphasic	47
4	Triphasic	28	Triphasic	38	Biphasic	36	Biphasic	36	Monophasic	31	Monophasic	38
5	Triphasic	36	Triphasic	33	Biphasic	62	Monophasic	32	Biphasic	41	Biphasic	47
6	Biphasic	40	Biphasic	42	Biphasic	42	Biphasic	40	Biphasic	45	Biphasic	38
7	Biphasic	45	Biphasic	43	Biphasic	58	Biphasic	52	Biphasic	47	Biphasic	46
8	Biphasic	32	Biphasic	36	Biphasic	35	Biphasic	37	Biphasic	31	Biphasic	32

*Vmax, maximum flow velocity*.

### Perioperation Outcomes

**Figure 3:**
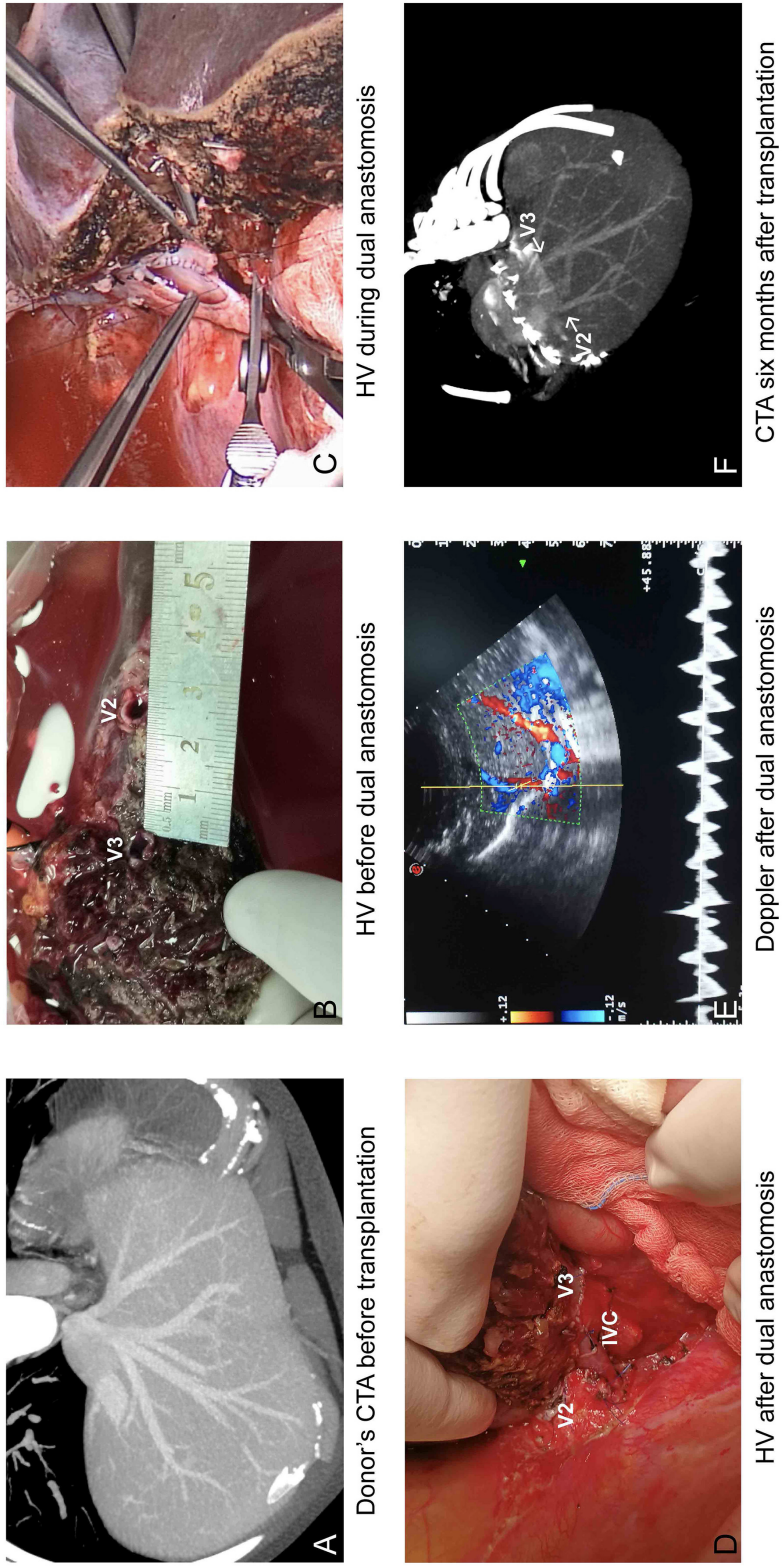
Operational results of modified dual hepatic vein anastomosis. **(A)** Donor's CTA before transplantation and **(B)** during transplantation showed two separated orifices in cut surface; **(C)** V2 was anastomosed to the common trunk of MHV and LHV, and V3 was anastomosed to longitudinal incision orifice in IVC. **(D)** HV after dual anastomosis. **(E)** Venous outflow was satisfactory on Doppler. **(F)** CTA at 6 months after liver transplantation.

For the eight patients using modified dual hepatic anastomosis, all of them have satisfactory venous flow after LDLT, and no HV-related complications were observed in the subsequent follow-up. Graft function recovered within 2 weeks. The median period for drainage tube indwelling was 15 days (7–37), and the details of abdominal drainage are summarized in Figure 3. The median time of hospital stay was 19 days (14–37). The Doppler US tests after LDLTs (postoperation days 7 and 14) showed fluent hepatic vein flow, and the peak velocities are summarized in Table 3. Among recipients, six (75%) patients have biphasic wave pattern of V2 and V3 at postoperation days 7 and 14. One patient have monophasic wave pattern of V2 and biphasic wave pattern of V3 at postoperation day 14. In addition, one recipient have monophasic wave pattern of both V2 and V3 at postoperation day 14. However, Up to the last follow-up, none of the patients had clinical symptoms caused by hepatic venous obstruction.

## Discussion

Adequate venous outflow has been recognized to be a vital element for the success of liver transplantation (14). Hepatic veins are characterized by low pressure and high flow rate as the draining basin of IVC. Anastomotic narrowing, twisting, or inadequate drainage of accessory veins may cause outflow obstruction and result in severe graft dysfunction, such as sepsis, jaundice and small-for size syndrome which worsen the prognosis of transplant patients (15, 16). Due to the anatomical variation of LHV, the size, relative location, and number of venous orifices at the cut surface are various which increase the difficulty of venous anastomosis in the left lateral segment graft LDLT (17). Nevertheless, vessel allografts procured from recipients and deceased donors may cause extra injury and increase reconstruction time (18). It is essential to find and adopt proper technique in different LHV variants to ensure fluent venous outflow in LDLT.

In this research, LLS graft donors were classified into three types according to the hepatic vein orifice variations at the cut surface (6, 19, 20). For type I with a single opening, hepatic venous anastomosis was performed using the stump of the middle and left hepatic veins. The right edge of the MHV is incised to enlarge the orifice to a dimension in keeping with the size of the graft hepatic vein. This technique fixes the graft on the wall of the IVC and helps prevent the graft from rotating. Emond et al. recommended the use of a triangular anastomosis to allow the graft to be fixed well on the wall of the IVC by forming a large anastomosis (21). LLS graft with type II variation can be effectively reconstructed using wedged unification. In LDLT, using left lateral segment graft with the typeIII variant, inadequate drainage of segment III will cause excessive bleeding during the operation (22). Hepatic venous outflow obstruction also leads to regurgitation of portal flow in the congested graft which make the remnant liver cannot meet the metabolic demands (23).

Venoplasty has been applied to form a single opening to facilitate the vein anastomosis in type IIIa (orifices distance <20 mm) (10, 24). For type IIIb (orifices distance >20 mm), interposition conduit using vessel grafts has been proven to have good functional results. Iliac artery is the preferable choice for its thick wall which can prevent collapse and ensures patency in low pressure flow (11, 17). Endarterectomized atherosclerotic artery allograft has been used as an alternative vessel material when other adequate vein graft material is unavailable (18). Veerankutty et al. reported a quadrangular patch venoplasty technique using iliac veins to reconstruct LHV in LDLT (25). Nevertheless, intractable shortage of vessel allograft from deceased donors make it hard to meet the need with a rapid increase of LDLT. Although autologous graft veins have also been applied for reconstruction of hepatic veins in LDLT (26), this strategy may cause additional damage to patients and cost more operation time. In addition, overlong (>20 mm) interposition conduit and endarterectomized vessel allograft are more likely to induce venous congestion. Obstruction of interposition conduit will not only lead to S3 congestion but also affect the V2 drainage which brings more risk for LLS grafts with thick V3 (R_V_ ≤ 1). Therefore, we introduced modified dual hepatic vein anastomosis technique for type IIIb variant LLS graft with R_V_ ≤ 1.

Dual hepatic vein anastomosis was adopted for grafts with two wide orifices which cannot form a common anastomotic stump. Two independent anastomosis can provide a stable dual-axis structure to prevent twisting or kinging. Reconstruction of V2 and V3, respectively, also prevent the whole LLS venous obstruction from interposition conduit congestion. However, traditional dual hepatic vein anastomosis were made between the graft HVs and the recipient MHV and LHV or RHV and the common stump of MHV/LHV (10). This anastomosis is required for size matching between the distance of graft hepatic vein orifices and the diameter of the recipient's IVC. In addition, separate hepatic vein anastomoses frequently lead to partial or complete occlusion during liver graft regeneration. Here, we introduced a modified dual hepatic vein anastomosis to improve the traditional surgical technique. V2 is anastomosed end-to-end to the left of the common stump of MHV and LHV while V3 is anastomosed end-to side to the longitudinal incision orifice in the anterior wall of IVC. The incision orifice should be located next to the middle line of IVC. This leaves enough space for graft regeneration and enlarges the range of LLS grafts selection for which orifice distance is slightly bigger than the length of IVC. Hepatic vein reconstruction was performed with 5–0 polydioxanone (PDS), and eversion suture was not suggested to maximize outflow size which helps to prevent venous obstruction.

In the LDLTs performed from September 2018 to December 2019, we identified 27 LLS grafts with two wide separate hepatic orifices at the cut surface. A common of eight children used modified dual hepatic vein anastomosis to reconstruct venous outflow. During the maximal follow-up period of 50 months, no HV complications occurred and all the eight children recovered smoothly. Although longer follow-up and larger sample size are required, the encouraging results in this research confirm the efficacy and safety of the modified dual hepatic vein anastomosis for type IIIb grafts with R_V_ ≤ 1.

In conclusion, our modified dual hepatic vein anastomosis technique provides a concise method for type IIIb grafts with R_V_ ≤ 1 to reconstruct venous outflow. For type IIIb LLS grafts with R_V_ > 1, conduit is also available considering that small V3 conduit occlusion has a limited effect on graft survival.

## Data Availability Statement

The raw data supporting the conclusions of this article will be made available by the authors, without undue reservation.

## Ethics Statement

Written informed consent was obtained from the individual(s), and minor(s)' legal guardian/next of kin, for the publication of any potentially identifiable images or data included in this article.

## Author Contributions

QX and PW designed and performed the surgery. YH collected the data and wrote the paper. MF and TZ participated in arterial anastomosis. BQ and JZhu analyzed the data. YL and JZha participated in donor operations. All authors contributed to the article and approved the submitted version.

## Funding

This study was supported by Shanghai Sailing Program (18YF1412700 [PW]), the Project of Medical Key Specialty of Shanghai Municipality (shslczdzk05801 [QX]), and the National Key Research and Development Program of China (2017YFC0908100 [QX]).

## Conflict of Interest

The authors declare that the research was conducted in the absence of any commercial or financial relationships that could be construed as a potential conflict of interest.

## Publisher's Note

All claims expressed in this article are solely those of the authors and do not necessarily represent those of their affiliated organizations, or those of the publisher, the editors and the reviewers. Any product that may be evaluated in this article, or claim that may be made by its manufacturer, is not guaranteed or endorsed by the publisher.

## Abbreviations

LLS: left lateral segment
LDLT: living liver transplantation
MHV: middle hepatic vein
LHV: left hepatic vein
IVC: inferior venous cave
US: doppler ultrasound
BA: biliary atresia
OTCD: ornithine transcarboxylase deficiency
GRWR: graft/recipient's body weight ratio
Rv: ratio of veins (V2/V3).
